# Augmenting osteoarthritis–fracture risk mitigation: leveraging digital health for precision prevention

**DOI:** 10.1097/JS9.0000000000003185

**Published:** 2025-08-21

**Authors:** Yuxia Yang, Meijuan Yuan, Qing Kong, Zhiwei Peng, Yucheng Zhang

**Affiliations:** a School of Medicine of Yangzhou University, Yangzhou, P. R. China; b Northern Jiangsu People’s Hospital, Affiliated to Yangzhou University, Yangzhou, P. R. China; c The Second Affiliated Hospital of Xuzhou Medical University, Xuzhou, P. R. China; d Department of Orthopaedics, Changshu Hospital Affiliated to Nanjing University of Chinese Medicine, Suzhou, P. R. China; e Yangzhou University, Yangzhou, P. R. China

*Dear Editor,*

We read with great interest the prospective cohort study by Wang *et al* examining the association between osteoarthritis (OA) and future fracture risk in the extensive UK Biobank dataset[1]. The findings of a significant association (adjusted HR 1.11, 95% CI 1.08–1.15; *P* < 0.0001), with falls mediating approximately 15% of this risk, provide crucial population-level evidence reinforcing the imperative need for enhanced fracture prevention strategies in OA patients. While the authors comprehensively establish this vital epidemiological link, we wish to expand the discussion on the implementation of preventive measures, specifically highlighting the transformative potential of digital health technologies (DHTs), advanced analytics, and computational modeling to operationalize these findings into scalable, personalized clinical actions.

Wang *et al* rightly conclude that preventing fractures in OA populations is essential. However, identifying which OA patients are at highest imminent risk remains a significant challenge within traditional clinical parameters. Here, DHTs offer a paradigm shift. Wearable inertial measurement units (IMUs) and smart insoles now enable continuous, real-world monitoring of gait kinetics, variability, and balance – parameters intrinsically linked to fall risk, the mediator accounting for ~16% of the OA–fracture association identified in this study. Coupled with machine learning algorithms, these DHTs can predict fall likelihood with increasing accuracy in free-living environments, providing objective, dynamic risk stratification far exceeding sporadic clinical assessments[2]. Integrating such sensor-derived biomechanical data with the rich clinical and demographic variables used by Wang *et al* (like OA severity, location, prior fall history, body mass index) could create highly refined, individualized risk prediction models, moving beyond population-level hazard ratios to pinpoint personal vulnerability.

The concept of the “digital twin,” a dynamic, personalized computational model simulating an individual’s physiological state and potential health trajectories, holds exceptional promise in this context[3]. As outlined in foundational work on predictive health modeling, these virtual replicas can assimilate multi-modal data streams: continuous biometrics from wearables, periodic imaging data quantifying joint degeneration and bone quality, electronic health record data, and genetic risk scores where available. Applying such a framework to OA patients, a digital twin could simulate personalized future fracture probabilities under different scenarios, facilitating truly precision prevention. Clinicians could virtually test the impact of targeted interventions (e.g., specific balance training intensity, adjustments to bone-active medication) before implementation, optimizing resource allocation toward those predicted to benefit most.

Wang *et al*’s work underscores the critical need for accessible, scalable interventions. Digital therapeutics (DTx) directly address this challenge[2]. Evidence-based, app-delivered exercise programs, tailored specifically for OA patients and incorporating proven principles for improving strength, proprioception, and balance, have demonstrated significant efficacy in improving pain and function[4]. Crucially, DHTs enable remote monitoring of adherence to these programs, a persistent barrier to effectiveness, and allow clinicians to track functional gains objectively through embedded sensors or patient-reported outcomes. This continuous feedback loop optimizes rehabilitation effectiveness, targeting the modifiable OA-related factors contributing to fracture risk beyond just falls.

Therefore, while Wang *et al* provide compelling epidemiological evidence for OA as a fracture risk factor, we emphasize that translating this knowledge into clinical impact necessitates embracing innovation[1]. DHTs provide the necessary infrastructure: wearable sensors enable objective, continuous risk stratification focused on gait and fall mediators; computational models like digital twins facilitate individualized risk forecasting; and DTx platforms deliver accessible, remotely monitored interventions targeting the modifiable drivers identified. The scale and granularity of datasets like UK Biobank validate these approaches. The future of fracture prevention in OA lies in integrating these evolving capabilities into structured care pathways, transforming population-level risk into personalized, actionable prevention strategies as a direct application of findings like those presented by Wang *et al*. Further research should prioritize feasibility studies and health economic assessments of such integrated digital approaches in diverse OA cohorts. (We ensure this article is compliant with the TITAN Guidelines)[5].

## Data Availability

Not applicable.
